# The stability and self-assembly of tri-calcium silicate and hydroxyapatite scaffolds in bone tissue engineering applications

**DOI:** 10.1186/s13036-025-00481-4

**Published:** 2025-02-17

**Authors:** Nima Beheshtizadeh, Amir Abbas Seraji, Behnam Azadpour, Sima Rezvantalab

**Affiliations:** 1 Department of Tissue Engineering, Faculty of Advanced Medical Sciences, Tabriz University of Medical Sciences, Tabriz, Iran; 2 Regenerative Medicine Group (REMED), Universal Scientific Education and Research Network (USERN), Tehran, Iran; 3 Department of Mechanical and Industrial Engineering, University of Toronto, Toronto, Canada; 4 Department of Polymer Engineering and Color Technology, Amirkabir University of Technology, Tehran, Iran; 5 Department of Chemical Engineering, Brigham Young University, Provo, UT 84602 USA; 6 Renewable Energies Department, Faculty of Chemical Engineering, Urmia University of Technology, Urmia, 57166‑419 Iran

**Keywords:** Bone scaffolds, Calcium silicate, Calcium phosphate, Hydroxyapatite, Rheology performance, Molecular dynamics simulations

## Abstract

The fabrication of scaffolds for bone tissue engineering (BTE) applications often involves the utilization of two distinct categories of biomaterials, namely calcium phosphates and calcium silicates. The selection of these materials is based on their biocompatibility, bioactivity, and mechanical characteristics that closely resemble those of natural bone. The present research examined the utilization of hydroxyapatite (HAP) and tri-calcium silicate (TCS), which are among the most commonly utilized materials in calcium phosphates and calcium silicates, in the context of bone scaffolding applications. A molecular dynamics simulation was conducted to investigate the impact of different concentrations of ceramic nanoparticles, when combined with sodium alginate (SA) hydrogel, on the fabrication of bone scaffolds.

The stability and self-assembly were assessed through several parameters, such as the solvent-accessible surface area (SASA), radius of gyration (Rg), radial distribution function (g(r)), root-mean-square deviation (RMSD), root-mean-square fluctuation (RMSF), hydrogen bonding, van der Waals, electrostatic, and total energies. The findings indicate that the addition of 10 wt% HAP and TCS to the SA hydrogel matrix results in a more compact, stable, and potentially less hydrated structure. Accordingly, the experimental validation of these simulation approved our in silico findings. Experimental rheology and mechanical properties evaluation validate our simulation results, indicating a superior characteristic of TCS10 and HAP10 inks and 3D-printed scaffolds among other composition ratios. This could potentially benefit the in vitro and in vivo performance of the scaffold and its interaction with cells. The aforementioned traits are considered fundamental for the successful execution of the scaffold in the field of BTE. The findings indicate that TCS samples exhibit superior properties when compared to HAP samples, specifically in terms of composition with SA hydrogel.

## Introduction

Numerous investigations have been carried out in the field of bone tissue engineering (BTE) with the objective of surmounting the associated obstacles. The primary aim of tissue engineering (TE) is to generate functional substitutes for damaged tissues. The replacement of bone defects that possess intricate structures with appropriate three-dimensional (3D) constructs has the potential to facilitate the regeneration of bone in the affected region. Hence, a variety of 3D scaffolds have been produced with the aim of providing a temporary framework for the growth of new tissue to replace damaged tissue [1].

The scaffold must possess certain attributes that facilitate the restoration of the original tissue’s structure and function subsequent to its replacement by the scaffold. The success of tissue regeneration is dependent on several attributes such as biocompatibility, biodegradability, bioactivity, mechanical properties, and pore size [2]. These attributes play a crucial role in facilitating cell adhesion and proliferation, as well as the diffusion of nutrients and gases. The efficacy of bone repair is heavily reliant on the scaffold’s structural and mechanical properties, thus necessitating the development of 3D constructs that can be implanted into anatomical defects [1].

A number of biomaterials have been utilized to fabricate porous scaffolds with precise control over morphology, pore size, and porosity [3, 4]. These biomaterials include bioactive glass, calcium phosphates, calcium silicates, and various polymers. In contemporary times, the production of bone scaffolds has diversified to encompass a range of biomaterials, some of which exhibit superior mechanical robustness, more stable self-assembling, and excellent biological behavior.

However, the fabrication of scaffolds for BTE often involves the utilization of two distinct categories of biomaterials, namely calcium phosphates and calcium silicates [5]. The selection of these materials is based on their biocompatibility, bioactivity, and mechanical characteristics that closely resemble those of natural bone [6]. Porous scaffolds derived from calcium phosphates and calcium silicates have the ability to serve as a structural support system for the proliferation of fresh osseous tissue [7].

The regulation of scaffold porosity is crucial in achieving a biomimetic architecture that mimics the natural bone structure, thereby facilitating osteogenesis by promoting the infiltration of osteogenic cells and the subsequent formation of new bone tissue [8]. The scaffold’s mechanical characteristics can be tailored to mimic those of the host bone, thereby enabling the scaffold to furnish the requisite support during the reparative phase.

Calcium phosphates, encompassing hydroxyapatite (HAP), tri-calcium phosphate (TCP), biphasic calcium phosphate (BCP), and other related materials, constitute a distinct group of substances [9]. The chemical composition of these materials bears resemblance to the mineral component of bone, rendering them highly appealing for employment in BTE. HAP is widely recognized as the most thermodynamically stable form of calcium phosphate and serves as the predominant mineral constituent in both osseous and dental tissues [10]. The material exhibits remarkable biocompatibility and osteoconductivity, facilitating the adhesion and proliferation of osteogenic cells and the genesis of fresh osseous tissue.

On the other hand, calcium silicates, namely wollastonite (CaSiO_3_), di-calcium silicate (Ca_2_SiO_5_), and tri-calcium silicate (Ca_3_SiO_5_, TCS), have been utilized in the field of BTE [11]. The aforementioned materials possess bioactive properties, which facilitate the induction of osteogenesis, or the formation of novel bone tissue. TCS has been the subject of extensive research, as evidenced by previous studies [12]. The production of Portland cement heavily relies on the utilization of TCS as a fundamental constituent. Upon exposure to water, a chemical reaction may occur with this substance, ultimately leading to the creation of calcium silicate hydrate [13].

The enhancement of mechanical strength can potentially be achieved through the consolidation and reinforcement of the microstructure of calcium silicate hydrate. According to recent research, there exist several indications that TCS possesses bioactive and biodegradable properties [14, 15]. The study by Pei et al. [15]. investigated the utilization of 3D printing technology in the production of TCS/mesoporous bioactive glass cement scaffolds, which were subsequently subjected to a curing process. The cement scaffolds were created by integrating the hydraulic properties of TCS with the notable biological features of bioactive glass. In their study, the researchers observed that their composition exhibited macropores that were linked in a three-dimensional manner, with pore sizes measuring 400 μm [15]. Additionally, the composition displayed a porosity of almost 70%, a mechanical compressive strength of approximately 12 MPa, and remarkable apatite mineralization capability [15]. The authors arrived at the conclusion that 3D-printed composite cement scaffolds, subjected to a curing process, exhibit suitability for the purpose of bone regeneration.

Incorporating TCS ceramic powders into fabricated scaffolds creates an alkaline environment that is conductive to the differentiation of stem cells into bone cells at low densities, as previously reported [16]. However, at high densities, the TCS nanopowders are unsuitable for cell attachment due to the hydrophobic nature of the resulting scaffolds [17]. However, the main question in preparing the bone constructs is remained as whether calcium phosphate scaffolds are more appropriate or calcium silicates. To the best of our knowledge, a few studies have compared these two groups of biomaterials in BTE applications. The multiple aspects of these biomaterials should be investigated to address this concern.

One of the fundamental underpinnings of biological and physical activity is rooted in molecular interactions [18], which arise from the configuration of macromolecular structures [19]. Currently, simulation techniques are considered valuable instruments for assessing and potentially forecasting the characteristics and efficacy of materials and structures that contain multiple components, as well as their interactions with other substances and surroundings [20]. In addition, simulations offer rational justifications for structures and phenomena that cannot be accessed experimentally. The utilization of a mathematical model prior to conducting experimental evaluations is a cost-effective approach that reduces the expenses associated with superfluous analyses.

The utilization of molecular dynamics (MD) simulations has evolved into an advanced methodology for establishing correlations between the structure and function of macromolecules [21]. MD simulations were employed to gain atomic-level insights into the interactions and bonds present in the composite scaffolds that were developed. Physiologically significant time intervals can be juxtaposed with the temporal duration of the simulation process. In the field of structural bioinformatics, a comprehensive understanding of the dynamic characteristics of macromolecules is capable of shifting the focus from analyzing individual structures to evaluating conformational ensembles [21].

**Fig. 1:**
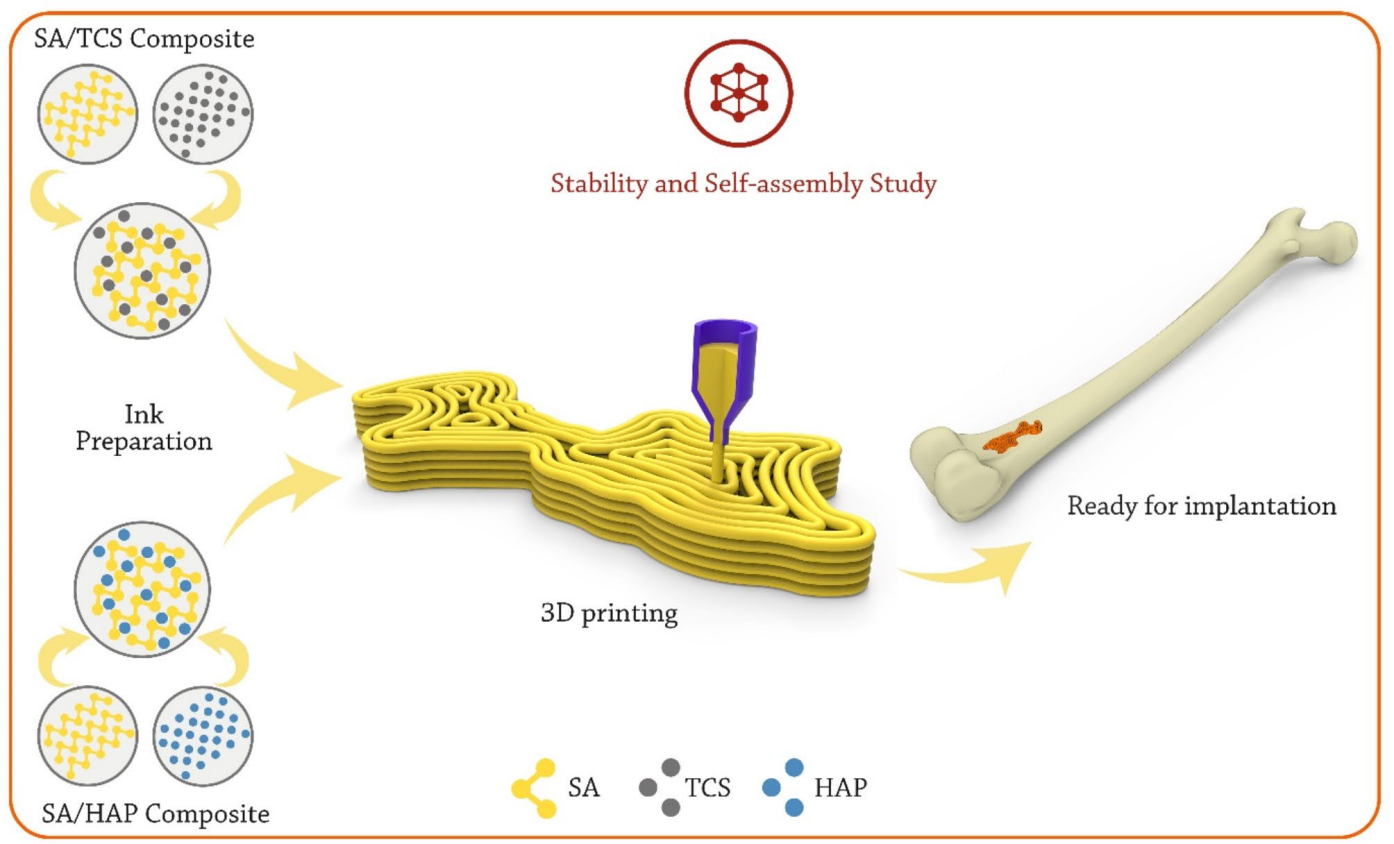
Developing SA/TCS and SA/HAP inks for preparing 3D bone scaffolds. Various complex structures would be accessible via this technique. The research question is that whether SA/HAP scaffolds or SA/TCS scaffold possess more stability and self-assembly features in this application

The present study employs atomic-scale simulations to investigate the differences among HAP and TCS, as most usage biomaterials of calcium phosphates and calcium silicates, respectively. MD simulation was utilized to optimize the density and concentrations of HAP and TCS in combination with sodium alginate (SA) hydrogel to fabricate the bone scaffolds. In addition, a variety of parameters associated with the self-assembly of biomaterials were considered, including solvent-available-surface-area (SASA), root-mean-square-deviation (RMSD), radius of gyration (Rg), H-bonds, root-mean-square fluctuation (RMSF), and radial distribution function (RDF, g (r)).

In addition, the electrostatic and van der Waals (vdW) energy levels, as well as total energy levels, are studied for evaluating the self-assembly of the HAP and TCS nanopowders in combination with SA hydrogel. The 3D printing technique was assumed for developing bioinks to prepare 3D bone scaffolds (Fig. 1). To validate our simulation findings, rheological behavior of the developed bioinks as well as mechanical performance of the 3D-printed scaffolds were analyzed through experimental evaluations. The findings presented provide significant understanding regarding the utilization of HAP and TCS nanopowders to enhance the efficacy of bone constructs.

## Materials and methods

### Molecular dynamics (MD) simulations

In the current research, we have developed mineral-polymer composites based on SA with TCS or HAP. To obtain comparable simulations, all simulations were based on weight percentages (0, 10, 20, 30, and 40%) of minerals in the total compositions. A hexagonal-type HAP (Ca_10_[PO_4_]_6_[OH]_2_) and orthorhombic tri-calcium silicate (Ca_3_SiO_5_) substrates were prepared using the Charmm-GUI nanomaterial modeler [22]. Polymer chains containing 5 monomers were prepared and preequilibrated with Avogadro software (version 1.2.0). The topology of the polymer structure was obtained using the Charmm-GUI Ligand Reader & Modeler [23] with the Charmm-36 m force field [24]. In the next step, all-atom molecular models with multiple molecules solvated in water were prepared using Charmm-GUI Multicomponent Assembler [25]. Table 1 outlines the number of components used in simulations.

**Table 1:** Details about the simulation models

	Number of molecules incorporated in simulations		Number of molecules incorporated in simulations	
Weight%	SA	HAP	SA	TCS
0	28	0	-	-
10	25	1	59	1
20	22	2	52	2
30	19	3	45	3
40	16	4	39	4
Simulation box	6 × 6 × 6 nm^3^		7 × 7 × 7 nm^3^	
Water models	TIP3P		TIP3P	
Salt	NaCl		NaCl	
Salt concentration	0.15 M		0.15 M	

Afterwards, using GROMACS 2020, the simulations were further equilibrated with Periodic boundary conditions (PBCs) in the EM (10 kJ/mole/nm minimum force), NVT (500 ps with 1 fs time steps) and NPT (500 ps with 1 fs time steps). The Particle Mesh Ewald (PME) [26] for long-range electrostatic interactions, the isotropic Parrinello-Rahman algorithm [27] for the pressure at 1 bar, and the Berendsen thermostat for temperature algorithms at 300 K were employed. The hydrogen bonds were constrained using the LINCS algorithm [28]. During all steps, the vdW and Coulomb interactions have been considered within a 1.4 nm cutoff radius. Finally, MD simulations were carried out for 100 ns with a 2 fs time step. Further analysis of the simulations was carried out using GROMACS software. Additionally, visualization of the models was performed via VMD software [29].

### Experimental analysis

Sodium alginate (SA), tricalcium silicates (TCS) and hydroxy apatite (HAP) were purchased from Sisco Research Laboratories Pvt. Ltd. Company, India. To produce a suitable ink for extrusion-based 3D printing, a precise quantity of TCS and HAP nanopowders were added to the SA solution. Multiple inks were produced with varying compositions of SA/TCS and SA/HAP to be tested via the rheology analysis. Table 2 shows the composition of each specimen.

To create the specimen No. 2, SA/TCS: 90/10, pour 2.16 g of SA into a beaker with 20 ml of distilled water and stir at 35 ℃ for an hour. Then, 0.24 g of TCS was gradually added to the solution to create the mentioned composite. The mixture was agitated at room temperature for 30 min to produce a homogenous ink ready to be put into the printer cartridge. All additional specimens with varying SA/TCS and SA/HAP concentration and solid/solution ratios were created using the same approach.

The rheological properties of the inks were assessed at 25 ℃ using an MCR-702 multi-drive rheometer (Anton Paar GmbH, Austria), equipped with a PP25 parallel plate of 25 mm diameter. The viscosity curves were produced using shear rate measurements, with a logarithmic scale with 1 s^− 1^ per data point, and a range of 0.001–100 s^− 1^. An amplitude examine was performed with a strain range of 0.01 to 100% at a constant temperature of 25 ℃ and a frequency of 1 Hz. 10 s was the duration of data collecting for each data point. Using a logarithmic scale ranging from 1 to 10,000 Pa, the storage and loss moduli (G′ and G′′) were determined by an amplitude sweep operating at 1 Hz. G′ and G′′ were quantified in the linear viscoelastic region (LVR) at plateau values.

The composite scaffolds were fabricated using an extrusion-based 3D printer (Abtin Teb Company, Iran). The fabrication process was similar to our previously fabricated bone scaffolds [30]. 3D-printed scaffolds were freeze-dried for 24 h in -80 ℃. As a means of measuring the compressive strength of specimens under loading conditions of 1 mm/min, the STM20 test machine (Santam Company, Iran) equipped with a 200 KN load cell was used. For this assessment, each sample was resized to dimensions of 7 × 7 × 6 mm^3^. The experiment was conducted in triplicate for each condition. A scanning electron microscope (SEM, Philips XL30: Eindhoven, The Netherlands) was used to examine the samples for their structure and composition.

**Table 2:** Developed bioinks with various compositions of SA/TCS and SA/HAP

Sample name	TCS	HAP	SA
SA / HAP 0 / TCS 0	0	-	100%
TCS 10	10%	-	90%
TCS 20	20%	-	80%
TCS 30	30%	-	70%
TCS 40	40%	-	60%
HAP 10	-	10%	90%
HAP 20	-	20%	80%
HAP 30	-	30%	70%
HAP 40	-	40%	60%

## Results and discussion

### Optimization of TCS content in SA hydrogel

**Fig. 2:**
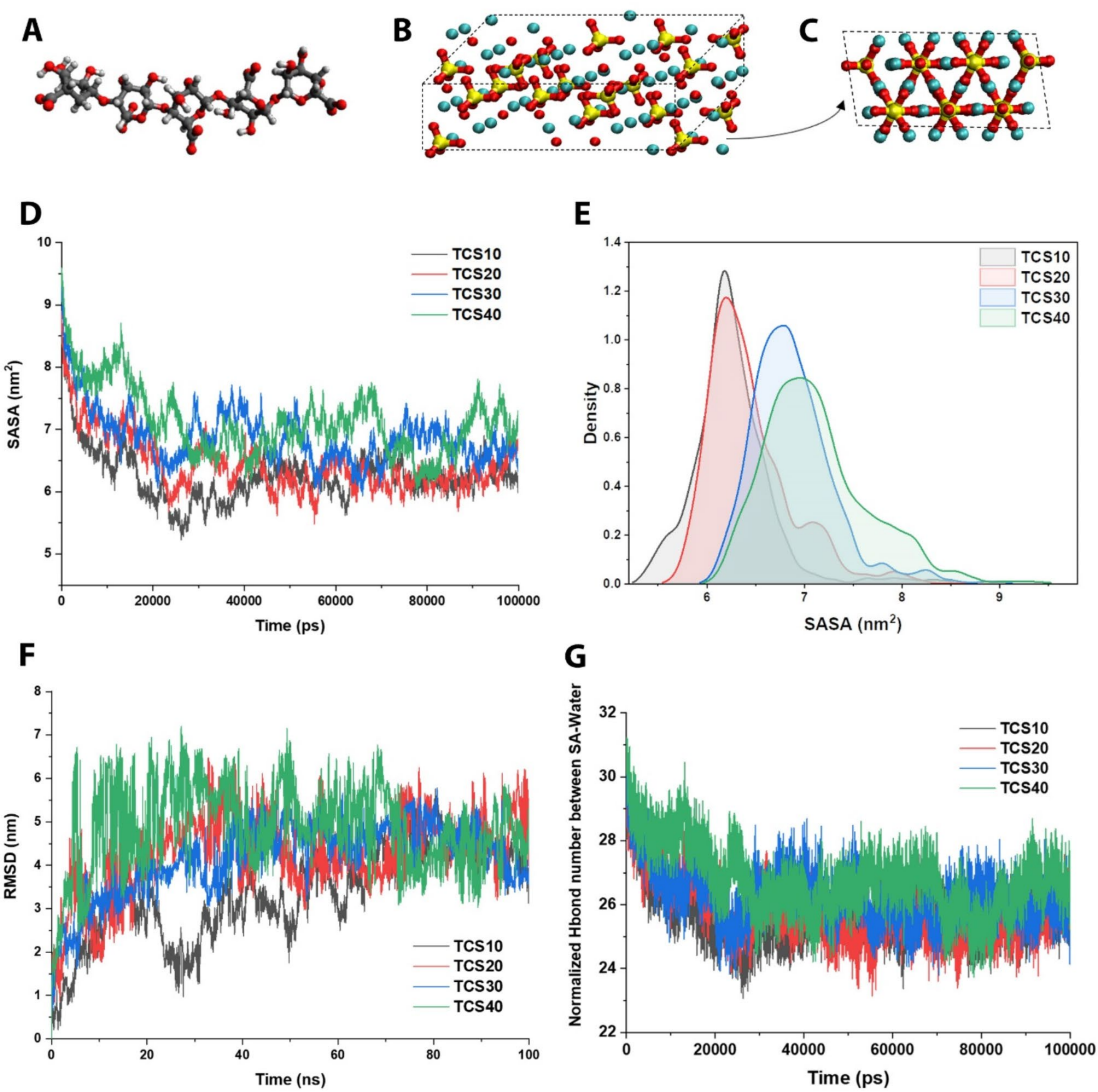
Analyzing the integration of TCS in SA hydrogel through SASA, RMSD, and H-bond (**A**) SA molecule; (**B**) TCS molecule; (**C**) side view of the TCS molecule; (**D**) SASA analysis fluctuations of the 10, 20, 30, and 40 wt% TCS in SA hydrogel composition; (**E**) SASA distribution diagram of the 10, 20, 30, and 40 wt% TCS in SA hydrogel composition; (**F**) RMSD analysis fluctuations of the 10, 20, 30, and 40 wt% TCS in SA hydrogel composition; and (**G**) normalized H-bond number diagram among SA-water molecules. All the SASA, RMSD, and H-bond analyses confirmed that 10 wt% TCS has the best and optimal state over the simulation

A comparable study was performed to evaluate the efficiency of TCS content (0, 10, 20, 30, and 40 wt%) in SA hydrogel. Figure 2A shows the SA molecule, while Fig. 2B shows the TCS molecule, and its side view is illustrated in Fig. 2C, which were used in the current MD simulation. Considering the effects of the various concentrations of the components in the composite materials, they should be optimized and engineered. Here, we optimized the TCS content in SA hydrogel through MD simulation.

The SASA diagram is a frequently employed instrument in MD simulations for comprehending the conductivity of molecules within a solvent milieu [31]. It refers to the extent of the surface area of a molecule that is available for interaction with a solvent. The SASA is frequently employed as a means of approximating the hydrophobic effect, a fundamental factor in the interactions of macromolecules [31].

Figure 2D and **E** present the aforementioned procedure, which can yield valuable insights pertaining to the conformational alterations exhibited by the molecule throughout the simulation. The lowest SASA value indicates that it has a dense structure that is not accessible to water molecules. The lowest amount of SASA is related to 10 wt% TCS, while in terms of distribution, the 10 wt% mode has the narrowest distribution, and the distribution peak is at the lowest number (around 6.2 nm).

Regarding the RMSD analysis, which is a widely utilized metric in MD simulations that serves to assess the dissimilarity in structure between two molecular configurations, it should be noted that the utilization of this technique is frequently observed in the evaluation of macromolecule structure stability in simulations. The RMSD is computed by taking the square root of the mean of the squared distances between the atoms of two given structures, following the optimal alignment of said structures [32].

The structures are commonly overlaid in a manner that reduces the RMSD, frequently through the alignment of the macromolecule structure’s backbone atoms. Recalling that a molecule’s stability and equilibration can be inferred from a low and consistent RMSD value, whereas a high or escalating RMSD value may suggest substantial conformational changes occurring within the molecule [33], it is important to note that both over time and on average, the best case in our simulation is related to 10 wt% TCS (Fig. 2F).

The analysis of hydrogen bonding (H-bond) is a vital component of MD simulations, as it significantly impacts the structure, stability, and function of biological molecules. A geometric criterion is commonly employed in MD simulations to define a hydrogen bond, which entails the use of a distance cutoff and an angle cutoff. The distance cutoff refers to the upper limit of the permissible distance that can exist between the donor atom, typically a nitrogen or oxygen atom, and the acceptor atom, which is typically a hydrogen atom that forms a covalent bond with a nitrogen or oxygen atom [34].

The angle cutoff refers to the minimal angle that is permissible between the atoms of the donor, hydrogen, and acceptor. Typical distance cutoff values range from 2.5 to 3.5 angstroms, while angle cutoff values typically range from 120 to 180 degrees [35]. However, it is important to note that these values may fluctuate depending on the particular investigation being conducted.

The analysis of hydrogen bonding within a MD simulation can yield diverse forms of information. The quantity of hydrogen bonds refers to the collective count of hydrogen bonds established by a molecule or a set of molecules within a specific timeframe. Also, the hydrogen bond lifetime refers to the temporal span during which a hydrogen bond persists, encompassing the interval between its formation and subsequent rupture [36]. This phenomenon has the potential to offer valuable insights regarding the stability of hydrogen bonds.

It is noteworthy that although the geometric criterion serves as a straightforward and widely employed approach for discerning hydrogen bonds, it constitutes an approximation and exhibits certain constraints. Although the presented model lacks consideration of factors such as the hydrogen bond strength and the unique chemical surroundings of the atoms, the lowest amount of hydrogen bonding of SA molecules with water indicates the stability of the formed assembly over time. Both over time and on average, the optimum content in our simulation is related to 10 wt% TCS (Fig. 2G).

**Fig. 3:**
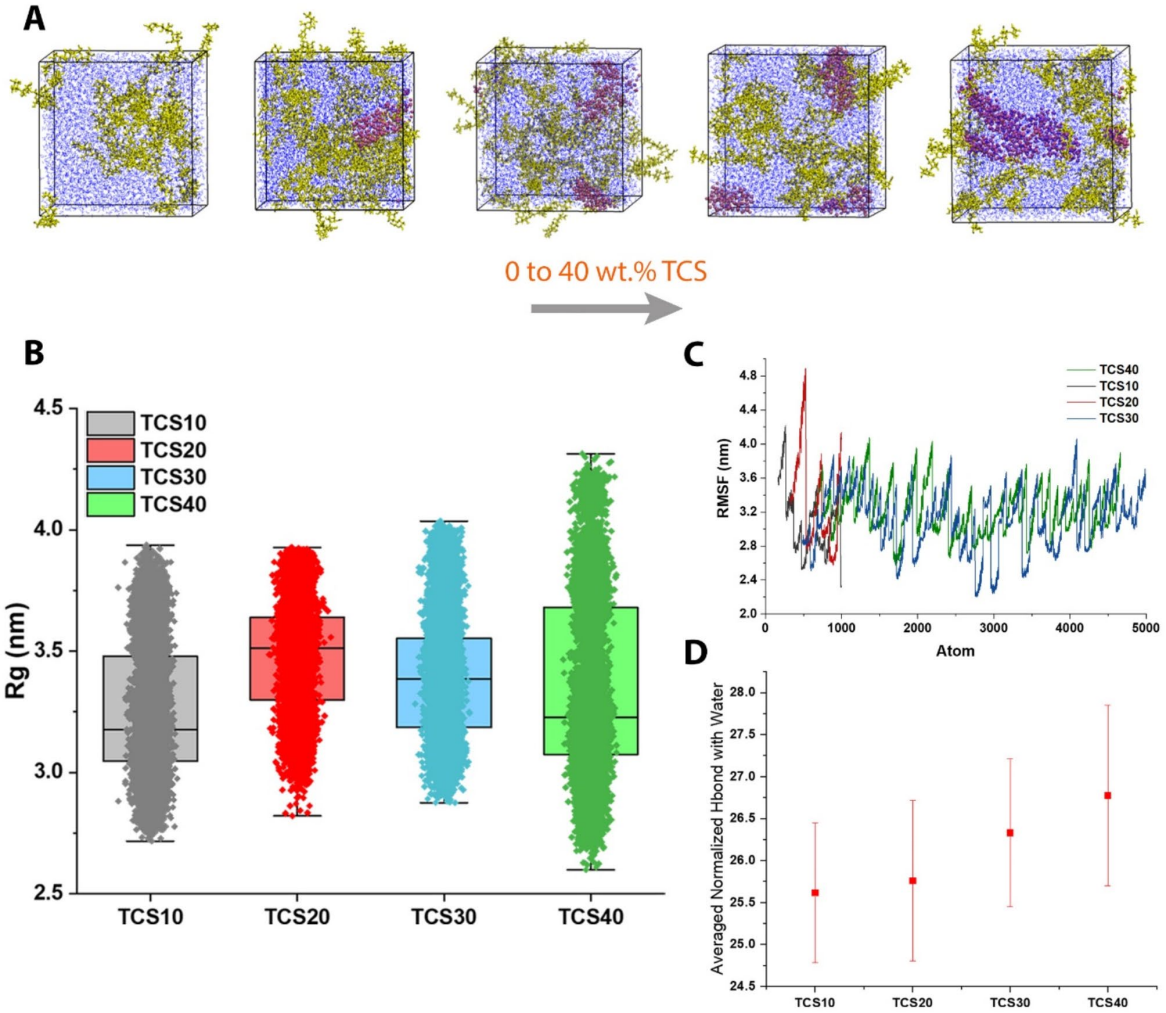
Analyzing the integration of TCS in SA hydrogel through Rg, RMSF, and H-bond (**A**) TCS molecules (purple) in the SA hydrogel (yellow), containing the water molecules (blue), showed the increasing of TCS content from 0 to 40 wt% in composition; (**B**) Rg analysis; (**C**) RMSF analysis; and (**D**) averaged normalized H-bond with water analysis. All the analyses confirmed that 10 wt% TCS has the best and optimal state over the simulation

The integration of TCS ceramic molecules with water and SA hydrogel molecules is depicted in Fig. 3A. The simulation illustrated that adding more TCS content resulted in more agglomeration in the composite system. Recalling that Rg is the radius of accumulation resulting from the accumulation of molecules, its results show the energy intensity between the molecules and the stability of the system. The technique offers insights into the spatial arrangement of mass within a given molecule, enabling deductions to be made regarding its stability and conformation. The radius of gyration can be defined as the root mean square distance between the center of mass and all constituent atoms within a molecule [37]. Stated differently, it calculates the mean displacement of each constituent with respect to the central point of the molecule while considering their respective masses.

The determination of Rg offers valuable insights into intermolecular energy intensity and system stability. A reduced value of Rg implies a condensed and densely packed molecule, indicating enhanced intermolecular associations and elevated stability [38]. Conversely, a greater value of Rg implies a molecule that is more expansive and pliable, thereby indicating diminished intermolecular associations and reduced stability. The energy intensity among molecules may be deduced by means of Rg, as it serves as an indicator of the molecule’s general shape and conformation. In the context of a polymer chain, a reduced Rg corresponds to a more tightly coiled and condensed conformation, indicating heightened intermolecular interactions and increased energy intensity. We analyzed the Rg among the interactions of TCS, SA, and water molecules. Results showed that TCS40 and TCS10 have the minimum Rg values, while TCS30 suffers from a high Rg amount (Fig. 3B).

RMSF is a metric utilized in MD simulations to quantify the typical deviation or fluctuation of atom positions from their respective average positions throughout the simulation. The biomolecular system’s flexibility and dynamics are elucidated by the information provided. The RMSF is commonly computed for individual atoms within a biomolecular system, yielding a profile of RMSF values for each atom [39]. This profile offers an analysis of the molecular regions’ flexibility and mobility. Elevated RMSF values are indicative of increased amplitude of motion and pliability, whereas diminished RMSF values imply heightened stability and inflexibility of the regions.

It is imperative to acknowledge that the significance of RMSF values is contingent upon the particular system and context. The degree of flexibility displayed by various biomolecules and simulation conditions may differ, resulting in varying levels of significance for RMSF values. Figure 3C shows the RMSF analysis results, validating the results obtained from previous analyses. Moreover, Fig. 3D summarizes the averaged normalized H-bond with water molecules, emphasizing that the minimum H-bond belongs to TCS10 samples. All the investigations positively correlate with each other, indicating that the optimal sample among the tested ones is dedicated to TCS10, which is more stable and performs higher energy bonds, lowering the solvent accessible surface area.

### Optimization of HAP content in SA hydrogel

**Fig. 4:**
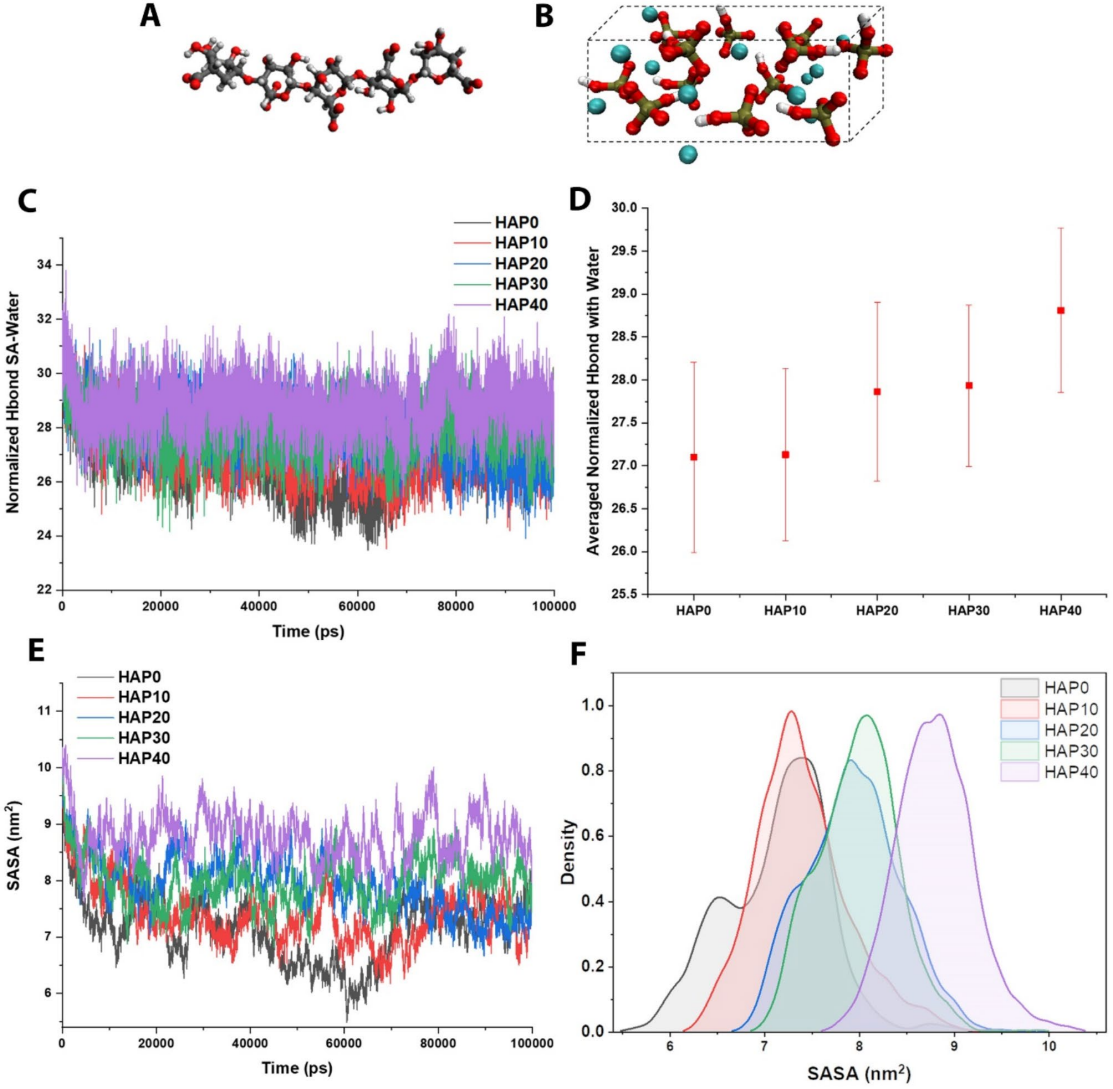
Analyzing the integration of HAP in SA hydrogel through H-bond and SASA (**A**) SA molecule; (**B**) HAP molecule; (**C**) normalized H-bond analysis among HAP and SA-water; (**D**) averaged normalized H-bond analysis among HAP and water; (**E**) SASA analysis fluctuations of the 0, 10, 20, 30, and 40 wt% HAP in SA hydrogel composition; and (**F**) SASA distribution diagram of the 0, 10, 20, 30, and 40 wt% HAP in SA hydrogel composition. All the H-bond and SASA analyses confirmed that 10 wt% HAP has the best and optimal state over the simulation

In this step, optimizing the content of HAP in SA hydrogel via MD simulations was performed, utilizing the SA molecule (Fig. 4A), HAP molecule (Fig. 4B), and water. Analyzing the H-bond among HAP, SA hydrogel, and water, the pure SA hydrogel possesses the lowest score, while it lacks the osteogenecity features of bone scaffolds (Fig. 4C). Hence, the second one, which is HAP10 (90 SA/10HAP composite), has the appropriate low H-bond score. This outcome was confirmed by the averaged normalized H-bond of HAP with water (Fig. 4D). Ignoring the pure SA hydrogel specimen, SASA reaches its lowest value in the case of HAP10, both in the fluctuation diagram (Fig. 4E) and the distribution peak diagram (Fig. 4F). The low values of SASA for the HAP10 show that the polymers are not spread towards the water and are densely packed together, so this sample is more stable.

**Fig. 5:**
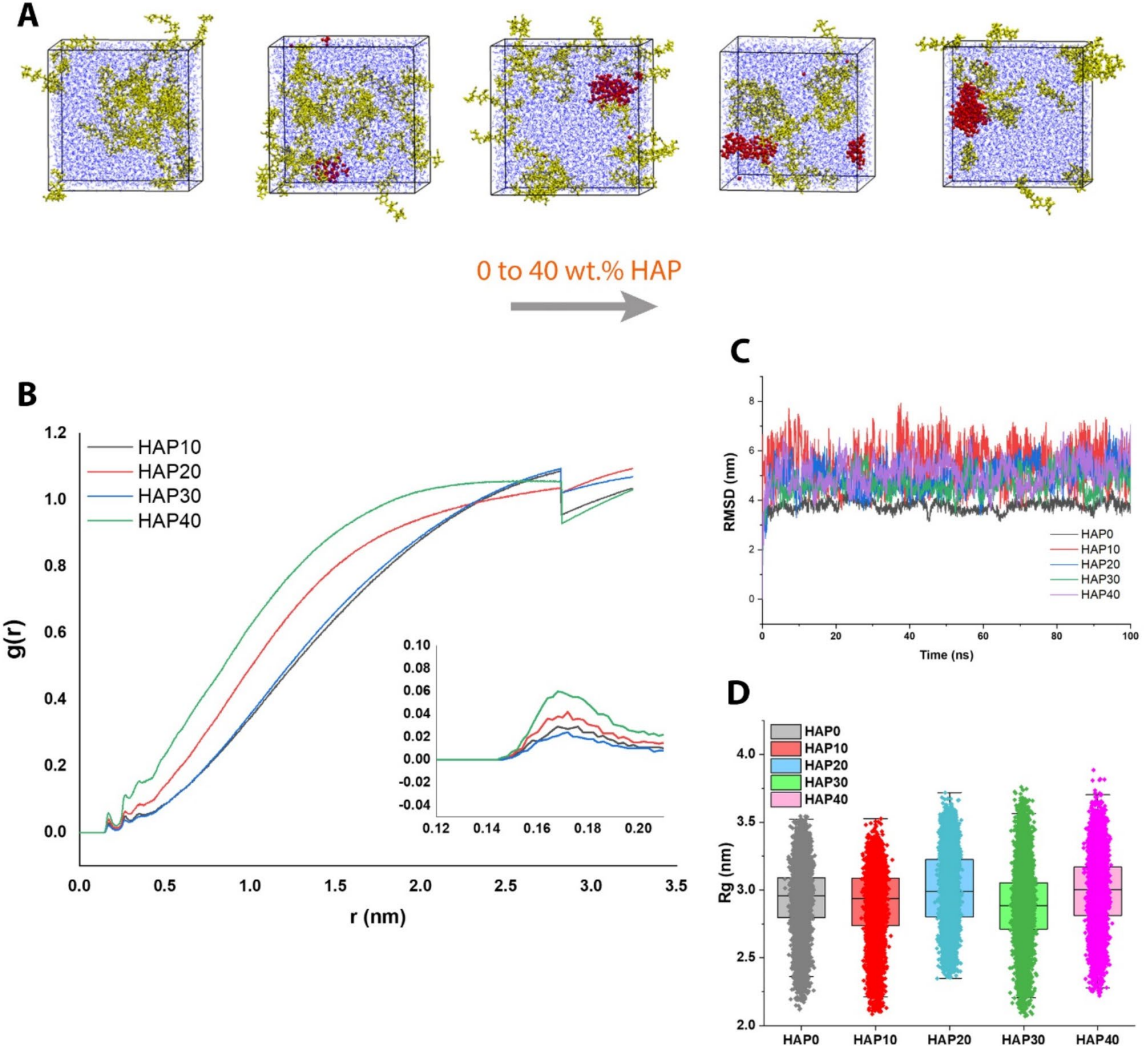
Analyzing the integration of HAP in SA hydrogel through g(r), RMSD, and Rg (**A**) HAP molecules (red) in the SA hydrogel (yellow), containing the water molecules (blue), showed the increasing of HAP content from 0 to 40 wt% in composition; (**B**) g(r) analysis; (**C**) RMSD analysis; and (**D**) Rg analysis. All the analyses confirmed that 10 wt% HAP has the best and optimal state over the simulation

The integration of HAP molecules (red) in the SA hydrogel (yellow), containing the water molecules (blue), is shown in Fig. 5A. Increasing the HAP content in the composite specimens enhances the agglomeration possibility, as shown in the simulation box. In terms of the radial distribution function, HAP10 has a uniform distribution in small amounts, while the rest of the percentages have a wide distribution and even two peaks (Fig. 5B). Moreover, Fig. 5C demonstrated the RMSD analysis, which shows a molecule’s stability and equilibration that can be inferred from a low and consistent RMSD value, whereas a high or escalating RMSD value may suggest substantial conformational changes occurring within the molecule. Furthermore, the radius of gyration reaches its lowest value at the end of the simulation for the HAP10 sample (Fig. 5D). In terms of the average radius of gyration, HAP10 and HAP30 are almost close to each other, while HAP30 has a lower score with a very small difference, as well as the distribution of values for HAP10 is lower than that for HAP30; hence, HAP is more stable.

### Comparison of TCS vs. HAP

The present investigation employed a particular methodology to examine the impact of diverse concentrations of TCS and HAP in SA hydrogel on multiple properties that are pertinent to the production of bone scaffolds. According to the findings, the fabrication of bone scaffolds is most effective when utilizing a concentration of 10 wt% TCS and, similarly, 10 wt% HAP. The aforementioned deduction is grounded on a number of significant parameters, namely SASA, radius of gyration, hydrogen bond formation, RMSD, and RMSF.

The SASA is a quantitative metric used to determine the extent of a biomolecule’s surface area that is available for interaction with a solvent [40]. A decrease in SASA observed at a 10 wt% concentration of TCS and HAP indicates a possible increase in the structural stability and compactness of the hydrogel. Enhanced mechanical stability is a desirable attribute in bone scaffolds. Also, the radius of gyration is a metric that quantifies the dispersion of atoms around the center of mass, taking into account their respective masses. A decrease in the radius of gyration at a 10 wt% concentration of TCS and HAP implies that the atoms are situated in closer proximity to the center of mass, thereby indicating a more condensed structure. This phenomenon has the potential to enhance the mechanical robustness and structural integrity of the scaffold.

The formation of hydrogen bonds is of utmost importance for maintaining the stability of various biological structures. The presence of a reduced number of hydrogen bonds at a 10 wt% concentration of TCS and HAP may indicate a lower level of hydration in the hydrogel. This could potentially have a positive impact on the mechanical characteristics of the scaffold and its cellular interactions. Also, RMSD is a metric utilized to assess the mean distance between the atoms, typically the backbone atoms, of aligned proteins. A reduced RMSD observed at a 10 wt% concentration of TCS and HAP suggests a higher degree of stability in the structure, thereby resulting in fewer conformational changes. This characteristic is advantageous for preserving the scaffold’s integrity. RMSF is a quantitative metric utilized to assess the degree of flexibility exhibited by a given structure. A reduced RMSF observed at a 10 wt% concentration of TCS and HAP indicates a decreased degree of flexibility in the scaffold. This may imply a comparatively more stable and rigid structure.

It is imperative to comprehend the underlying reasons as to why the concentration of 10 wt% TCS and HAP may be affording these advantageous characteristics. HAP is a naturally-occurring mineral form of calcium apatite that predominates in human bones as the primary calcium source. The purpose of integrating it into the SA hydrogel is to simulate the physiological bone milieu and enhance osteogenic cell proliferation and differentiation. At a concentration of 10 wt%, an equilibrium appears to exist between the advantages of HAP integration and the preservation of the hydrogel’s characteristics.

Similarly, TCS has been used in bone scaffolds to affect the osteogenecity of the cells seeded on the scaffolds. The use of TCS in SA hydrogel can influence a range of properties that are crucial for the production of effective bone scaffolds. The optimal concentration of TCS will depend on the specific requirements of the scaffold, such as its mechanical strength, porosity, degradation rate, and ability to stimulate bone regeneration. However, studies showed that at a content of 10 wt% of TCS, the physical and mechanical properties are in a higher state [30].

The concentration at which HAP/TCS is present can potentially offer an optimal level of structural stability, allowing for the emulation of the bone environment. However, it is crucial to ensure that the hydrogel network remains undisturbed and is not overly impacted by the presence of HAP or TCS. Elevated levels of HAP/TCS may result in aggregation or non-uniform dispersion, thereby impeding the integrity of the hydrogel matrix and compromising its desirable characteristics. Preserving the scaffold’s capacity to undergo deformation and subsequent recovery in response to stress is crucial for its physiological role.

**Fig. 6:**
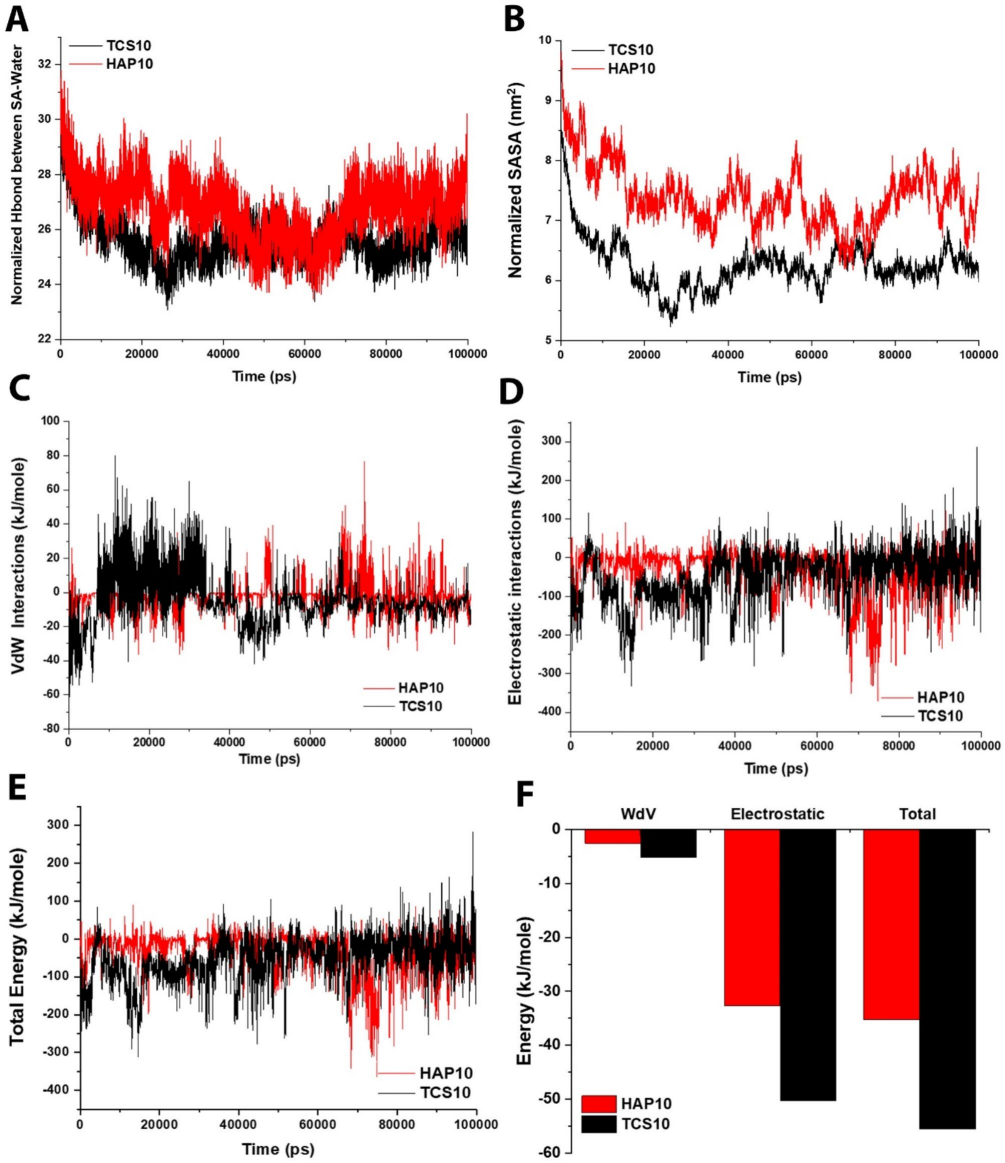
A comparison among TCS and HAP samples in SA hydrogel (**A**) Normalized H-bond between TCS10/HAP10 and SA and water; (**B**) Normalized SASA of TCS10 and HAP10 samples; (**C**) Vdw interactions of TCS10 and HAP10 samples; (**D**) Electrostatic interactions of TCS10 and HAP10 samples; (**E**) Total energy of TCS10 and HAP10 samples; and (**F**) Energy diagram of TCS10 and HAP10 samples. All the results indicated that the TCS10 sample is more stable than HAP10 and is suggested to be used in bone scaffolding applications

The reduced quantity of hydrogen bonds observed at a 10 wt% concentration of HAP/TCS may indicate a decreased level of hydration. This could potentially confer advantages on the mechanical characteristics of the scaffold and its cellular interactions. Excessive hydration of a scaffold may result in inadequate support for cellular proliferation, whereas insufficient hydration may lead to hindered nutrient transport and cellular migration. The results indicate that a structure with a 10 wt% HAP/TCS concentration exhibits lower RMSD and RMSF, which implies greater stability and reduced flexibility. The implementation of this approach may prove advantageous in preserving the structural soundness of the scaffold and facilitating a conducive milieu for cellular proliferation.

The comparable parameters between the two modes are the number of hydrogen bonds per chain unit (Fig. 6A) and the amount of SASA per chain unit (Fig. 6B). For the number of dehydrogenations per unit of SA with water, it can be seen that in the case of HAP, it is more connected with water. For SASA, HAP has more interaction with water. In terms of energy, the lower the Gibbs free energy of the self-assembly, the more stable and spontaneous the process. Hence, the sample containing 10 wt% TCS is more stable than the HAP sample (Fig. 6C-F).

Accordingly, it can be inferred that the utilization of a SA hydrogel with a 10 wt% HAP/TCS concentration exhibits a harmonious amalgamation of characteristics that are advantageous for the production of bone scaffolds. However, utilizing the TCS is more suggested in this study than HAP due to its stability and self-assembly parameters. Nevertheless, the aforementioned are conjectures derived from MD simulations, and it would be imperative to conduct empirical verification to substantiate these findings. It would be of interest to conduct an investigation into the temporal and physiological variations of these properties.

### Experimental analysis

**Fig. 7:**
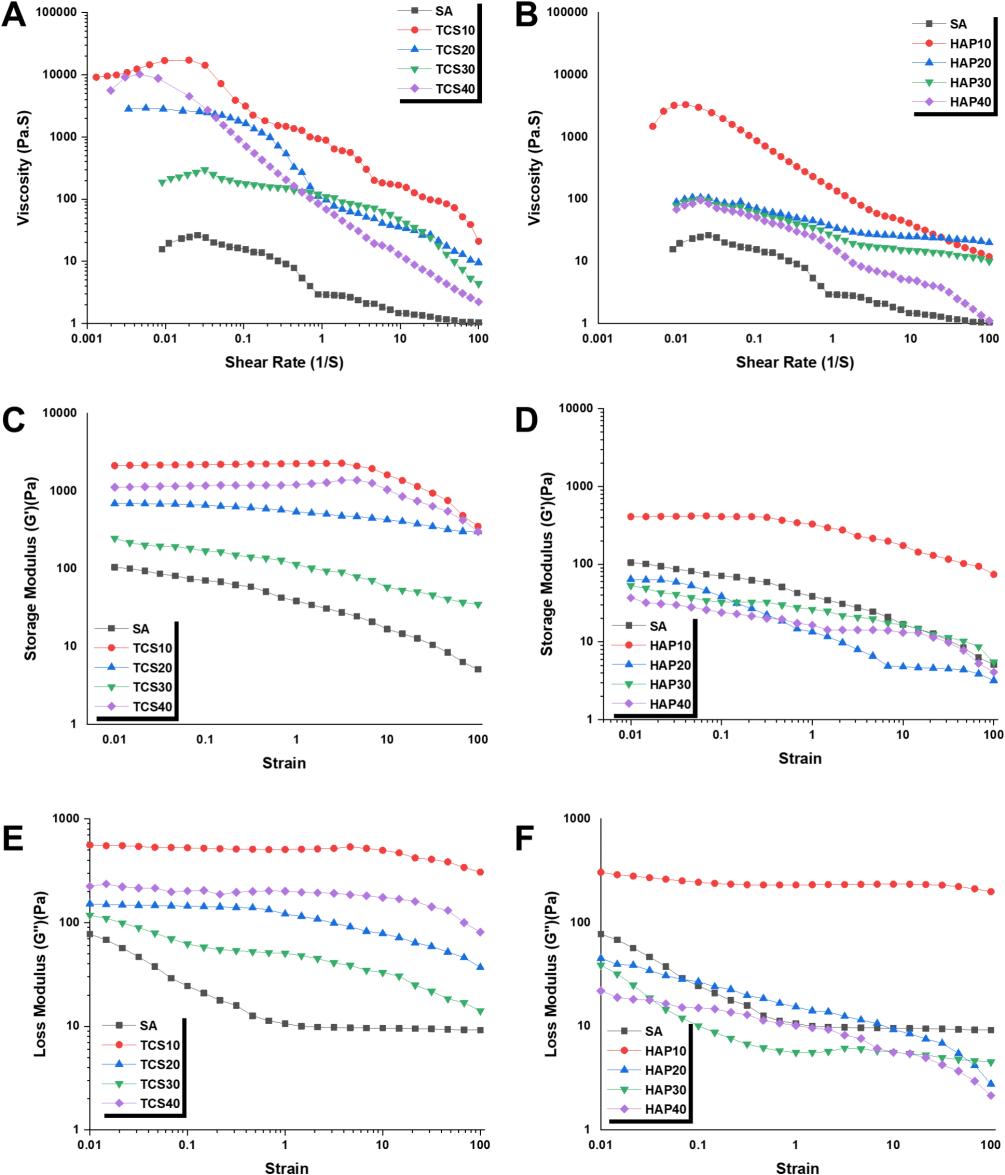
Rheological performance of various inks prepared for 3D printing: (**A**) Viscosity (Pa. s) vs. Shear rate (s^− 1^) for TCS specimens; (**B**) Viscosity (Pa. s) vs. Shear rate (s^− 1^) for HAP specimens; (**C**) Storage Modulus (G’) (Pa) vs. Strain for TCS specimens; (**D**) Storage Modulus (G’) (Pa) vs. Strain for HAP specimens; (**E**) Loss Modulus (G’’) (Pa) vs. Strain for TCS specimens; (**F**) Loss Modulus (G’’) (Pa) vs. Strain for HAP specimens. TCS10 and HAP10 has the best scores in all obtained rheology diagrams, while TCS10 possess higher scores vs. HAP10

In order to evaluate the accuracy and precision of obtained in silico results, we performed experimental analyses including the rheology test of bioinks and the mechanical performance test of 3D-printed scaffolds. Figure 7 shows the rheology performance analysis for various developed bioinks (see Table 2). This analysis includes viscosity (Pa. s) vs. shear rate (s^− 1^) for TCS (Fig. 7A) and HAP (Fig. 7B) specimens, storage modulus (G’) (Pa) vs. strain for TCS (Fig. 7C) and HAP (Fig. 7D) specimens, and finally loss modulus (G’’) (Pa) vs. strain for TCS (Fig. 7E) and HAP (Fig. 7F) specimens. TCS10 and HAP10 has the best scores in all obtained rheology diagrams, while TCS10 possess higher scores vs. HAP10.

Following a thorough assessment of the rheological properties of the formulated inks (Fig. 7), TCS10 and HAP10 with a SA/TCS and SA/HAP composition ratio of 90/10 were chosen for scaffold fabrication. These inks have suitable printability characteristics, with a viscosity exceeding 100 Pa⋅ s throughout a broad shear rate range of 0.001–100 s^− 1^. Moreover, these inks exhibited an appropriate storage modulus (G′ > 1000 Pa for TCS10 and G′ > 500 Pa for HAP10) and loss modulus (G′′ > 500 Pa) throughout a strain range of 0.01–100. The capacity to maintain printability over time is another advantage of these inks, while other low-viscosity inks deteriorated within a few hours.

**Fig. 8:**
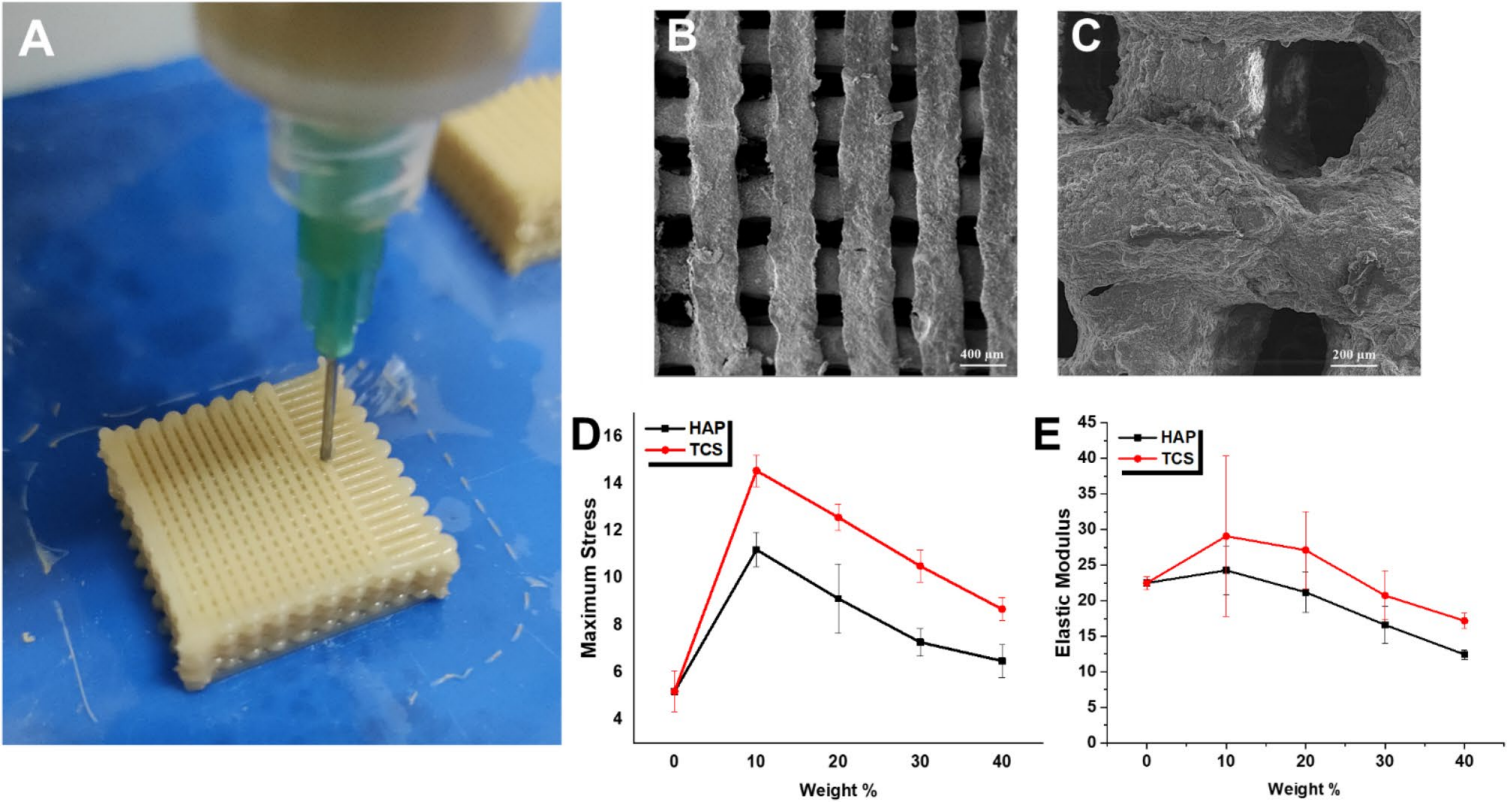
3D-printed scaffolds and their mechanical performance: (**A**) 3D printing procedure on TCS10 specimen via a bioink-filled cartridge assembled on 3D printer; (**B**) SEM analysis of 3D-printed TCS10 specimen; (**C**) SEM analysis of 3D-printed HAP10 specimen; (**D**) Maximum stress (MPa) diagram for various TCS and HAP specimens; (**E**) Elastic modulus (MPa) diagram for various TCS and HAP specimens. Diagrams showed that TCS10 and HAP10 specimens have the higher score in mechanical performance, while TCS10 possess higher score than HAP10 specimens

This study used extrusion-based 3D printing as an appropriate technique for generating polymer/ceramic composite scaffolds (Fig. 8A). A 3D linked cubic CAD model of 20 layers was designed with 300 μm micropores (Fig. 8B and **C**), suitable for the survival, proliferation, and differentiation of multiple cells. TCS10 with 14.55 MPa has the highest maximum stress among SA/TCS scaffolds, while HAP10 possesses 11.20 MPa as the strongest scaffold among SA/HAP scaffolds (Fig. 8D). Moreover, while pure SA scaffolds have 22.48 MPa elastic modulus, TCS10 and HAP10 scaffolds possess 29.07 and 24.26 MPa, respectively (Fig. 8E).

All these findings indicate that our experimental data well-verified obtained in silico MD simulation results, as the mechanical performance of a product, depends strongly on bonding between its various molecules. Various parameters of stability and self-assembly were evaluated in silico, including the SASA, Rg, g(r), RMSD, RMSF, H-bond, and Vdw, electrostatic, and total energies. Results showed that the incorporation of 10 wt% HAP and TCS in the SA hydrogel matrix seems to yield a denser, more stable, and possibly less hydrated architecture, which may have advantageous implications for the mechanical characteristics of the scaffold and its cellular interface. Rheological and mechanical tests validate our MD simulation findings and emphasized that the density, self-assembly, and stability of the scaffolds’ intra-architecture is a vital parameter for their performance in macro-scales.

The aforementioned characteristics are deemed essential for the efficacious implementation of the scaffold in the realm of bone tissue engineering. However, the comparison of TCS10 and HAP10 samples suggested the superiority of TCS samples with a 10 wt% content in composition with SA hydrogel. Nevertheless, it would be imperative to conduct preclinical tests in the animal body to further evaluate these simulation outcomes in order to substantiate these findings.

## Conclusions

This study investigated employing HAP and TCS as two of the most commonly used materials of calcium phosphates and calcium silicates in bone tissue engineering applications. An MD simulation was performed to study the effect of various concentrations of the mentioned ceramic nanoparticles in composition with SA hydrogel on the construction of bone scaffolds. Various parameters of stability and self-assembly were evaluated, including the SASA, Rg, g(r), RMSD, RMSF, H-bond, and Vdw, electrostatic, and total energies. Results showed that the incorporation of 10 wt% HAP and TCS in the SA hydrogel matrix seems to yield a denser, more stable, and possibly less hydrated architecture, which may have advantageous implications for the mechanical characteristics of the scaffold and its cellular interface. However, the comparison of TCS10 and HAP10 samples suggested the superiority of TCS samples with a 10 wt% content in composition with SA hydrogel, indicating the superiority of calcium silicate components to the calcium phosphate ones in terms of stability, self-assembly, and mechanical performance. The aforementioned characteristics are deemed essential for the efficacious implementation of the scaffold in the realm of bone tissue engineering. Nevertheless, it would be imperative to conduct preclinical tests in the animal body to further evaluate these simulation outcomes in order to substantiate these findings.

## Data Availability

All the data that support the findings of this study are available in this manuscript.

## Abbreviations

BTE: Bone tissue engineering
H-bond: Hydrogen bonding
HAP: Hydroxyapatite
MD: Molecular dynamics
RDF, (g(r)): Radial distribution function
Rg: Radius of gyration
RMSD: Root-mean-square deviation
RMSF: Root-mean-square fluctuation
SA: Sodium alginate
SASA: Solvent-accessible surface area
3D: Three-dimensional
TCS: Tri-calcium silicate
vdW: Van der Waals
